# Supplementary figures and images for: Assessing the extinction risk of Veracruz cycads
Source: Camb Prism Extinct. 2025 Mar 19;3:e7. doi: 10.1017/ext.2025.5 (PMC12034500; doi:10.1017/ext.2025.5)

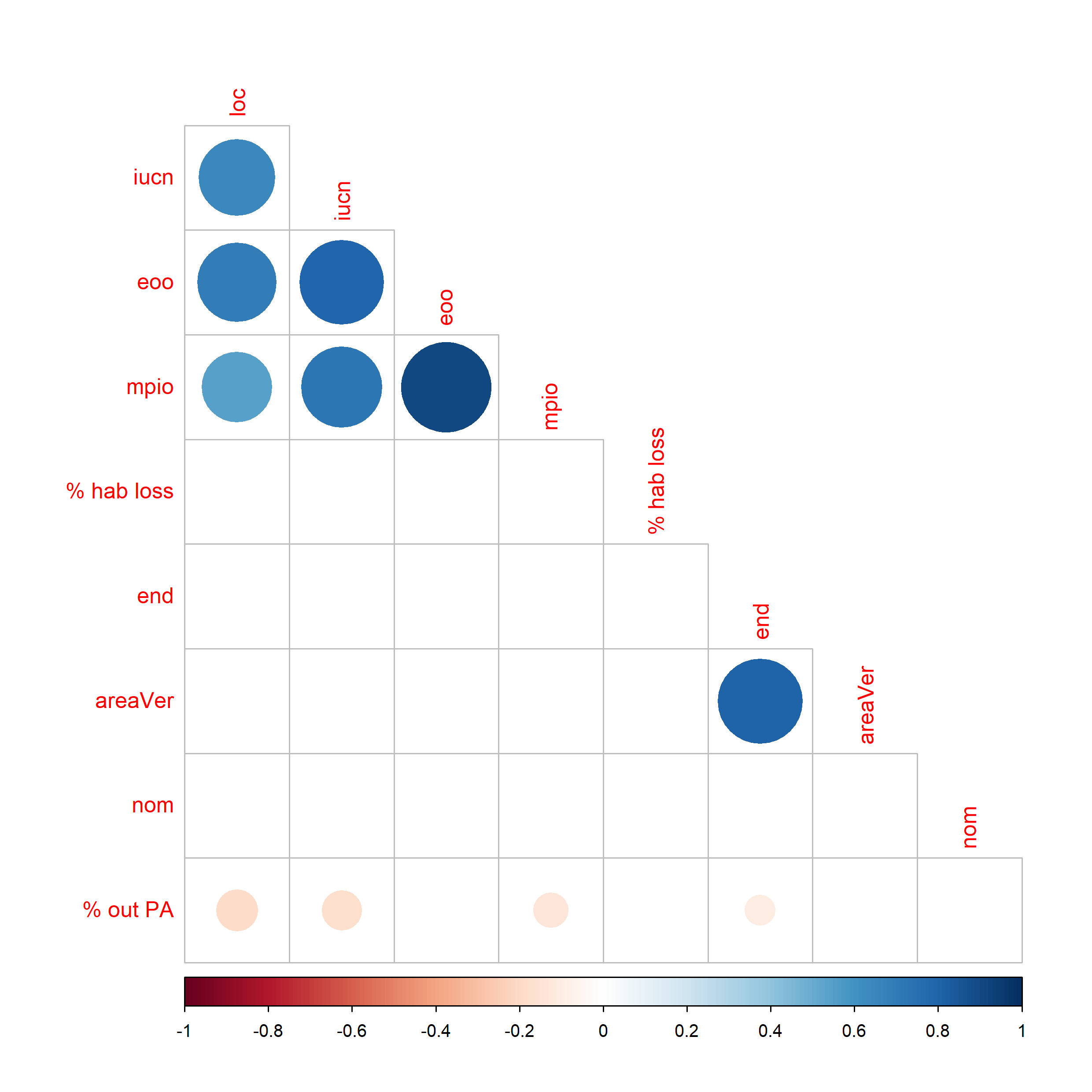

Supplement: Gómez Díaz supplementary material 1 — Gómez Díaz supplementary material [file S2755095825000051sup001.tif]
