# Supplementary material for: Assessing the extinction risk of Veracruz cycads
Source: Camb Prism Extinct. 2025 Mar 19;3:e7. doi: 10.1017/ext.2025.5 (PMC12034500; doi:10.1017/ext.2025.5)
Supplement: Gómez Díaz supplementary material 2 — Gómez Díaz supplementary material [file S2755095825000051sup002.docx]

**Supplementary figure S3**

Figure S3. Correlation among the variables used to create the Conservation Prioritization Index (CPI).
