# Supplementary material for: Assessing the extinction risk of Veracruz cycads
Source: Camb Prism Extinct. 2025 Mar 19;3:e7. doi: 10.1017/ext.2025.5 (PMC12034500; doi:10.1017/ext.2025.5)
Supplement: Gómez Díaz supplementary material 4 — Gómez Díaz supplementary material [file S2755095825000051sup004.docx]

**Supplementary text S2.** Keywords in English and Spanish to search studies on demography, population ecology, and population genetics of the species of Zamiaceae in Veracruz

English

1. Demography

2. Population ecology

3. Population genetics

4. Species ecology

5. Genetic diversity

6. Population dynamics

7. Reproductive biology

8. Conservation genetics

9. Population structure

10. Ecological genetics

Spanish

1. Demografía

2. Ecología poblacional

3. Genética de poblaciones

4. Ecología de especies

5. Diversidad genética

6. Dinámica poblacional

7. Biología reproductiva

8. Genética de la conservación

9. Estructura poblacional

10. Genética ecológica
